# Platelet‐Rich Plasma Versus Hyaluronic Acid and Vitamin C With Microneedling in the Treatment of Atrophic Acne Scars: A Comparative Study

**DOI:** 10.1111/jocd.71050

**Published:** 2026-07-14

**Authors:** Mai A. Samir, Amin Amer, Amin Sharobime, Alaa Mohamed, Rana Ehab

**Affiliations:** ^1^ Department of Dermatology, Venereology and Andrology, Faculty of Medicine Zagazig University Zagazig Egypt; ^2^ Department of Dermatology, Venereology and Andrology National Research Center Cairo (NRC) Giza Egypt; ^3^ Department of Dermatology, Venereology and Andrology Abou Hammad Central Hospital Sharkia Egypt

**Keywords:** atrophic acne scars, Dermapen, hyaluronic acid, microneedling, platelet‐rich plasma

## Abstract

**Background and Aim:**

Acne is a chronic inflammatory disease of the pilosebaceous units. Inflammatory acne may result in various kinds of scars, which can have a substantial influence on a patient's social and relationship life. The treatment of acne scars is challenging. To achieve optimal cosmetic results, a multimodal approach is required for scar treatment. The study evaluated the effectiveness and safety of microneedling with non‐crosslinked hyaluronic acid and vitamin C versus platelet‐rich plasma with microneedling for treatment of atrophic acne scars.

**Methods:**

This study included 34 patients (17–43 years) with atrophic acne scars. Dermapen was applied bilaterally, with platelet‐rich plasma on the right side and non‐crosslinked hyaluronic acid plus vitamin C on the left, over four sessions at three‐week intervals.

**Results:**

According to Goodman and Baron's qualitative acne scar grading system, both sides showed a significant improvement after treatment (*p* = 0.005, *p* < 0.0001 for each group). However, the response was significantly higher on the left side (vitamin C and hyaluronic acid) compared to the right side (PRP).

**Conclusions:**

We conclude that Dermapen with PRP or hyaluronic acid plus vitamin C improved acne scars, with better results for hyaluronic acid and vitamin C.

## 1 Introduction

Acne is a chronic inflammatory disease of pilosebaceous units with various clinical presentations, including comedones, erythematous papules and pustules, less frequently nodules, and deep pustules. Inflammatory acne causes various types of scars due to abnormal collagen degradation during the healing process [1].

Atrophic scarring, which occurs in 80%–90% of cases, is caused by collagen degradation at the dermal level [2] and is classified into three types: icepick (60%–70%), boxcar (20%–30%), and rolling scars (15%–25%) [3].

Acne scar treatments include chemical peels, microneedling, dermal abrasion, micro‐dermal abrasion, laser treatments, punch techniques, and combination therapies. Treatment options for hypertrophic scars and keloids include silicone gel, intralesional steroids, cryotherapy, and surgery [3].

Microneedling uses fine needles to create controlled skin injuries without damaging the epidermis, promoting wound healing by releasing growth factors and breaking down hardened scar strands for revascularization [4]. Furthermore, skin needling improves absorption of topical agents through a clear channel on the top layer of the skin, such as the platelet‐rich plasma (PRP), which contains autologous growth factors such as platelet‐derived growth factor (PDGF), epidermal growth factor, vascular endothelial growth factor (VEGF), and transforming growth factor β (TGF‐β) [5].

Hyaluronic acid (HA) is used routinely for hydrodynamic volume replacement of the extracellular matrix to reduce the clinical effects of aging. In the case of native non‐cross‐linked hyaluronic acid, it has been proven that the proliferative and metabolic activity of the cutaneous fibroblast has been increased, so it seems to be effective in treating atrophic acne scars [6].

Vitamin C (Vit C) has important physiologic effects on skin by promoting collagen biosynthesis, inhibiting melanogenesis, preventing radiation‐induced damage, and accelerating wound healing. Also, as an antioxidant, it effectively removes free radicals, leading to wound site improvements and potential benefits in treating atrophic acne scars [7].

The aim of this study was to compare the efficacy and safety of combined PRP with microneedling in comparison with microneedling with non‐cross‐linked HA and Vit C. The comparison method used in this study to assess the synergistic benefits of microneedling in combination with several agents is novel. Although each of these techniques has shown effectiveness on its own for skin rejuvenation, there is a lack of clinical studies directly comparing their combined effects.

## 2 Patient and Methods

This comparative split‐face study was conducted on 34 cases with atrophic post‐acne scars of different severities, determined clinically based on the typical appearance of skin lesions. Patients were selected from the outpatient clinics of the dermatology department. The study was approved by the local Institutional Review Board after all patients provided written informed consent. Because of the split‐face design, all patients were informed before enrollment that the two sides of the face might show unequal degrees of improvement. The consent process included an explanation of the possible cosmetic concern related to side‐to‐side differences, the expected transient adverse effects, and the plan for clinical follow‐up. Patients were assessed at each session and at follow‐up for adverse effects, patient concern, and clinically unacceptable asymmetry. If a persistent cosmetically unacceptable asymmetry occurred after completion of the final assessment, corrective treatment of the less‐improved side using the clinically more effective modality would be offered when appropriate.

Patients presented with a history of keloid formation, hypertrophic scars, immunosuppression, photosensitivity or current use of photosensitive medication, pregnancy, bleeding disorders, thrombocytopenia, patients having an active skin infection such as herpes simplex or systemic disease such as diabetes mellitus, collagen disease, oral isotretinoin use, radiofrequency, or laser skin resurfacing treatments in the preceding 6 months, and use of topical retinoids in the preceding 2 weeks were excluded from the study.

To assess scar types and severity, all patients underwent a history‐taking and dermatological examination using Goodman and Baron's qualitative global scarring grading system. It classifies post‐acne scarring according to clinical visibility and severity, ranging from mild scars that may be covered by makeup or facial hair to moderate scars that are not easily covered but may flatten with stretching, and severe scars that are not flattened by manual stretching [8].

Patients received dermapen with PRP on the right side of the face and dermapen with Vit C and HA on the left side (split face study).

### 2.1 Preparation of PRP

We collected 10 cc of autologous blood from each case. We obtained PRP and platelet‐poor plasma (PPP) by centrifuging whole blood with EDTA at 900 *g* for 5 min, then at 2000 *g* for an additional 15 min to separate from red blood cells. In the end, approximately 1–2 mL of PRP was gathered.

### 2.2 Preparation of Vitamin C and Hyaluronic Acid

For the left side of the face, 1 mL of 15% vitamin C and 1 mL of 1% non‐cross‐linked hyaluronic acid were prepared for each treatment session. The total volume applied to the left side per session was 2 mL. The hyaluronic acid preparation was a non‐cross‐linked, low‐viscosity HA serum rather than a cross‐linked filler; therefore, filler‐related rheological parameters such as gel stiffness were not applicable. The treatment was not administered by intradermal injection. Instead, HA was applied topically during and after microneedling to facilitate delivery through the microchannels created by the Dermapen, while vitamin C was applied after microneedling.

### 2.3 Procedure

A lidocaine/prilocaine cream was applied to the acne scar under occlusion for 45–60 min under aseptic conditions. Then, skin microneedling was performed using a dermapen (Dr. Pen Ultima A6 Professional Microneedling Pen; Bjheyetec Electronic Technology Co., Guangzhou, China) with a needle length of 1.5–2 mm and speed level 4. After sterilization with alcohol, 1–2 mL PRP was applied to the right side of the face and 1 mL of HA serum was applied to the left side of the face, followed by needling by moving the dermapen over the areas affected by acne scars six times in the four directions (vertically, horizontally, diagonally right, and left) without pressing. To reach the base of the scar, the skin was stretched perpendicular to the dermapen movement. Hyaluronic acid serum, or PRP, was applied during and after needling, while Vit C was applied after needling.

#### 2.3.1 Post Procedure Care

After each session, topical antibiotics, sunscreen, and moisturizer were prescribed. All patients were instructed to minimize sun exposure and informed that the face might look swollen and erythematous after the procedure, which could last for 2 or 3 days.

All patients received four sequences of treatment with an interval of 3 weeks.

#### 2.3.2 Clinical Assessment

Photographs were obtained using I phone 8+, 12‐megapixel camera at baseline, before the session, and at the end of treatment. The assessment was done 12 weeks after the final session. Two independent dermatologists assessed clinical results, blinded to treatments, using the quartile grading scale (QGS) and Goodman and Baron's qualitative scar grading system to determine improvements. The results were assessed using a QGS as follows: poor improvement below 25%, good improvement between 25% and 49%, very good improvement between 50% and 74%, excellent improvement over 75% [9].

### 2.4 Patient Satisfaction

At the end of treatment, the patients were asked to assess their degree of improvement as unsatisfied, satisfied, and very satisfied.

Side effects, such as infection, edema, erythema, herpes simplex, and post‐inflammatory hyperpigmentation, were noted at each session.

### 2.5 Statistical Analysis

Data were collected, coded, tabulated, and analyzed using IBM SPSS Statistics version 26.0. Quantitative variables were tested for normality and presented as mean ± standard deviation or median and range, as appropriate. Categorical variables were presented as frequencies and percentages. Because each patient received both treatment modalities in a split‐face design, paired statistical tests were used for side‐to‐side comparisons. Within‐side pre‐ and post‐treatment changes in Goodman and Baron's ordinal grades were assessed using the Wilcoxon signed‐rank test. Between‐side comparisons of ordinal clinical response categories were analyzed using paired non‐parametric testing. McNemar's test was used for paired categorical adverse‐event comparisons when applicable. Chi‐square or Fisher's exact test was used only for independent categorical variables. A *p*‐value < 0.05 was considered statistically significant.

**FIGURE 1:**
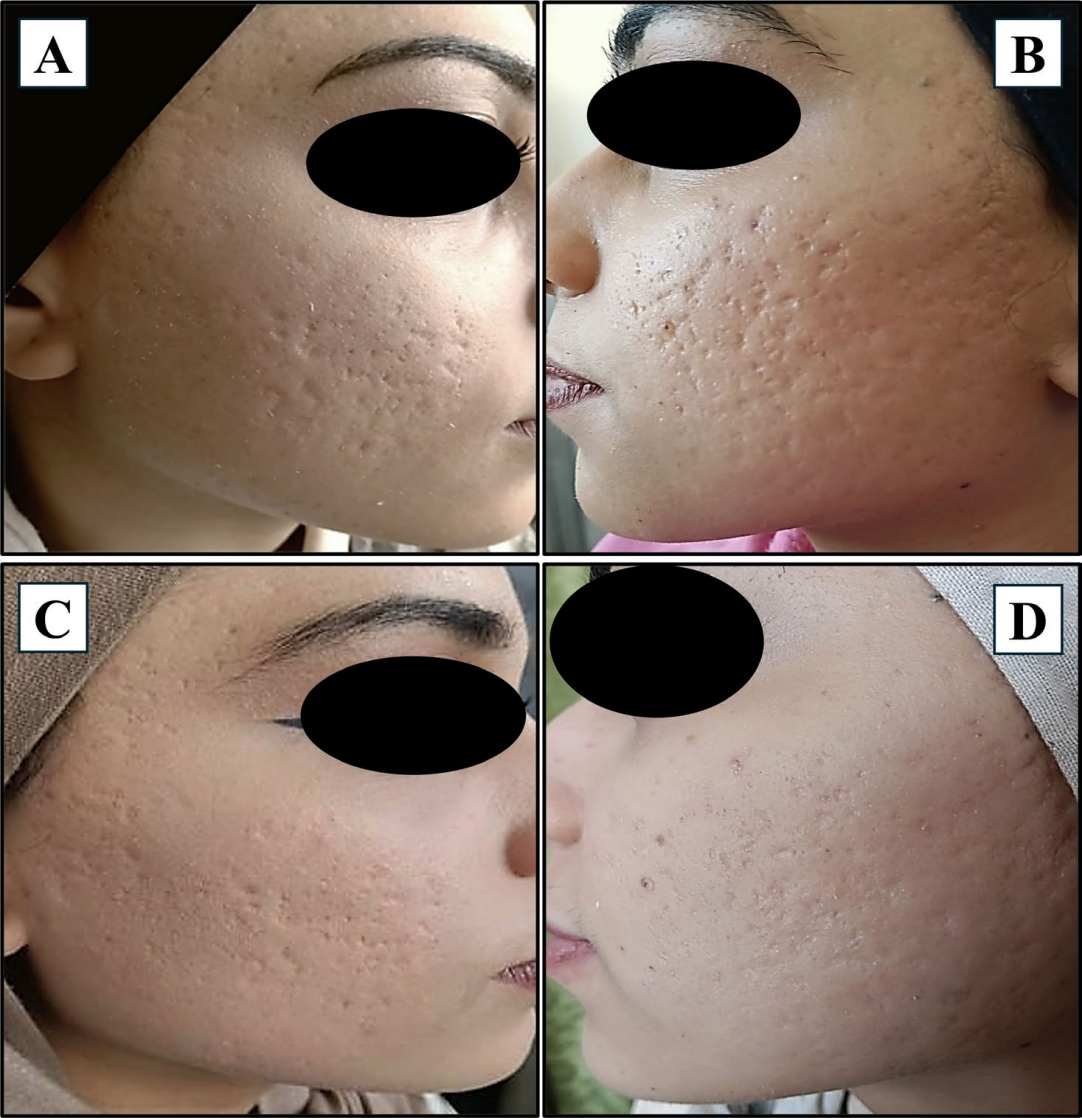
28‐year‐old female with severe atrophic acne scarring before treatment (right A and left B), showing good improvement on the right side after dermapen combined with PRP (C) and excellent improvement on the left side after dermapen combined with vitamin C and hyaluronic acid (D).

## 3 Results

This study included 34 patients who completed their sessions aged between 17 and 43 years with a mean age of 26.85 ± 5.75. Twenty‐eight patients were female, and six patients were male. Twenty‐two cases had a positive family history of acne scars. All patients had Fitzpatrick skin types ranging from II to V (Table 1). Regarding scar morphology, rolling scars were observed in 23 patients (67.6%), ice‐pick scars in 22 patients (64.7%), and boxcar scars in 10 patients (29.4%). Because several patients had more than one scar subtype, scar types were recorded as non‐mutually exclusive categories. Mixed scar patterns were present in 21 patients (61.8%).

**TABLE 1:** Baseline demographic and clinical characteristics of patients.

Variable	NO	%
**Age**		
Mean ± SD	26.85 ± 5.75	
Median (range)	26.5 (17‐43)	
**Sex**		
Females	28	(82.4%)
Males	6	(17.6%)
**Family history**		
Yes	22	64.7
No	12	35.3
**Skin photo types**		
II	6	17.6
III	19	55.8
IV	8	23.5
V	1	2.9
**Previous treatment**		
Yes	19	55.9
No	15	44.1
**Type of acne scar** ^**a**^		
Ice‐pick	22	64.7
Rolling	23	67.6
Boxcar	10	29.4
Mixed‐type scars	21	61.8

^a^ Patients may have more than one type of acne scar; mixed scars indicate patients with more than one scar subtype.

According to Goodman and Baron's qualitative acne scar grading system, both sides showed a significant improvement after treatment (*p* = 0.005, *p* < 0.0001 for each group). However, the response was significantly higher on the left side (Vit C and HA serum side) compared to the right side (PRP side) (Table 2).

**TABLE 2:** Comparison of therapeutic response according to clinical grading of Goodman and Baron between both groups before and after treatment.

	Skin micro needling combined with PRP side		Skin micro needling combined vitamin C and hyaluronic acid serum side		*p* ^a^
	No.	%	No.	%	
**Before treatment**					
Mild	8	23.5	5	14.7	0.65
Moderate	19	55.9	21	61.8	
Severe	7	20.6	8	23.5	
**After treatment**					
Mild	13	38.2	22	64.7	0.028 (S)
Moderate	17	50.0	12	35.3	
Severe	4	11.8	0	0.0	
*p* ^b^	0.005 (S)		< 0.0001 (HS)		

*Note:* *p* value < 0.05 is considered statistically significant (S). *p* value < 0.001 is considered statistically highly significant (HS).

^a^ Chi square test.

^b^ Wilcoxon ranked signed test.

According to the QGS, there was a significant difference after treatment in comparing both sides (*p* < 0.001). There was an excellent response in 18 cases (52.9%), a very good response in 10 cases (29.4%), and a good response in 6 cases (17.6%) on the side treated with HA serum and vit C in comparison to the side treated with PRP; 5 cases (14.7%) showed an excellent response, 10 cases (29.4%) showed a very good response, and 19 cases (55.9%) showed a good response (Figure 1; Table 3).

**TABLE 3:** Comparison of both treatment modalities for post acne scar according to quartile grading scale.

Quartile grading scale	Studied group				*p* ^a^	Post hoc test
	Skin micro needling combined with PRP side		Skin micro needling combined with vitamin C and hyaluronic acid serum side			
	*N*	%	*N*	%		
Excellent	5	14.7%	18	52.9%	0.001 (HS)	P1 = 0.008 P2 = 1 P3 = 0.001
Very good	10	29.4%	10	29.4%		
Good	19	55.9%	6	17.6%		

*Note:* *p* value < 0.001 is considered statistically highly significant (HS). P1: excellent response on PRP side vs. excellent response on Vitamin c and Hyaluronic acid serum side. P2: very good response on PRP side vs. very good response on Vitamin c and Hyaluronic acid serum side. P3: good response on PRP side vs. good response on Vitamin c and Hyaluronic acid serum side.

^a^ Chi square test.

As regards patients' satisfaction on the left side, 58.8% were very satisfied, 35.3% were satisfied, and 5.9% were not satisfied, while for the right side, 38.2% were very satisfied, 41.2% were satisfied, and 20.6% were not satisfied, with no statistically significant difference (*p* = 0.11).

Our patients experienced erythema and edema on both treated sides of the face that subsided within 2 to 3 days of the sessions, without a statistically significant difference between the sides. Table 4. The pain was tolerable in most patients.

**TABLE 4:** Side effects of treatment between right and left sides.

Side effects	Studied groups				*p* ^a^
	Skin micro needling combined with PRP side		Skin micro needling combined with vitamin C and hyaluronic acid serum side		
	*N*	%	*N*	%	
Erythema	20	58.8	16	47.05	0.33
Edema	17	50	19	55.88	0.63

*Note:* *p* value ≥ 0.05 is statistically non‐significant.

^a^ Chi square test.

Although the HA/Vit C side showed a significantly greater therapeutic response than the PRP side, no clinically unacceptable cosmetic asymmetry, deformity, or treatment‐related disfigurement was observed during the study period. The observed side‐to‐side difference was limited to greater scar improvement on the HA/Vit C side. No patient requested corrective intervention during follow‐up.

Age, gender, skin phototype, and scar type did not correlate significantly with the patients' degree of improvement on either side, as assessed by the QGS.

## 4 Discussion

The treatment of acne scars remains challenging, and the aim of treatment was to make the scars less obvious. Acne scars can cause psychological distress. As a result, such patients continually search for a rapid, non‐invasive, or minimally invasive method to treat scars [10, 11, 12].

Hence, the present study aimed to assess the efficacy and safety of microneedling for the treatment of post‐acne scars, using platelet‐rich plasma on the right side of the face and vitamin C and hyaluronic acid serum on the left side.

In our study, the combination of microneedling and PRP was found to be effective in treating acne scars on the right side in most cases. By QGS, 19 cases (55.9%) had a good response (25%–50%), 10 cases (29.4%) had a very good response (50%–75%), and 5 cases (14.7%) had an excellent response (> 75%).

Some studies have investigated the efficacy of microneedling combined with PRP. In agreement with the study by Asif et al. [13], who showed that there was a 62.20% improvement in the combined group (PRP and microneedling) compared to a 45.84% improvement with microneedling only in the treatment of atrophic acne scars. Similarly, studies by Fabbrocini et al. [14] and Ismail et al. [15] found that combining PRP with microneedling is more effective than microneedling alone for treating acne scars.

Skin needling creates micropunctures that break collagen bundles with subsequent induction of new collagen deposition, elastin, and vascularization in the papillary dermis to treat several skin conditions, such as decreasing the appearance of fine lines and wrinkles, skin laxity, and scarring [13, 16].

Platelet‐rich plasma has been used as an adjuvant treatment for atrophic post‐acne scars due to the presence of various growth factors like PDGF, VEGF, TGF‐β, and insulin growth factor‐1, which are released by concentrated platelets' α‐granules. PRP acts synergistically with growth factors induced by skin needling and intensifies the wound healing response [13, 17, 18].

In our study, combined microneedling and topical vit c and HA serum were found to be effective in the treatment of acne scarring on the left side in most cases. By QGS, 6 cases (17.6%) had a good response (25%–50%), 10 cases (29.4%) had a very good response (50%–75%), and 18 cases (52.9%) had an excellent response (> 75%).

Bano et al. [19] conducted a study comparing microneedling alone versus microneedling combined with hyaluronic acid for post‐acne scars in 60 patients, with 3 sittings at 3‐week intervals. The results showed that most patients had moderate improvement in both groups; however, more patients in the microneedling with hyaluronic acid group achieved “good” and “very good” outcomes by the end of treatment.

The higher results may be attributed to the use of combined microneedling with non‐cross‐linked HA and Vit C. As vitamin C is found to improve and promote tissue repair and neocollagenesis. It acts as a cofactor for the proline and lysine hydroxylases that stabilize the collagen molecule tertiary structure, and it also promotes collagen gene expression [20].

Also, some studies have investigated the efficacy of combined microneedling with Vit C. In 2014, Chawla [21] showed an excellent response in 18.5% of PRP‐treated cases, in comparison to only 7% who received Vit C treatment, according to a physician's assessment. Also, karim et al. [22] found an excellent response in 3 patients with PRP versus an excellent response in 1 patient with vitamin C for treating atrophic acne scars.

On the contrary, our results were higher than these results, which may be related to the combination of HA with Vit C. The precise mechanism of HA has been shown to accelerate tissue repair and wound healing [23]. In addition, it is believed to have anti‐inflammatory and bio‐stimulatory effects and may interact with various cell membrane receptors to trigger other signaling pathways [24].

In the current study, both sides of the face showed significant improvement of acne scars, but the improvement was significantly higher in the vit C and HA sides (*p* value = 0.028) according to the Goodman and Baron global score system (2006).

On the contrary, Amer et al. [25] found that microneedling with PRP (84.4%) yielded better results than microneedling with HA serum (82.9%); however, the difference was statistically insignificant (*p* > 0.05). This variation may be attributed to the use of a combination of non‐cross‐linked HA and Vit C.

As regards side effects in both sides of the face, the facial skin was red and swollen; however, in all patients, the redness and swelling subsided within 2–3 days of the sessions. Previous studies revealed similar side effects [14, 26, 27, 28, 29].

In conclusion, dermapen combined with either PRP or HA serum and Vit C showed satisfactory results and helped in the improvement of deep acne scars, but HA serum and Vit C combination gave better results. The present study has some limitations. First, the sample size was relatively small, which may limit the statistical power and generalizability of the findings. Second, the split‐face design, although useful for reducing interindividual variability, may raise cosmetic concerns if one facial side responds better than the other. However, no clinically unacceptable asymmetry requiring corrective treatment was observed in this study. Third, the follow‐up period was relatively short, and longer follow‐up is needed to determine the durability of improvement and late adverse effects. Fourth, assessment depended mainly on clinical grading scales and photographic evaluation; therefore, future studies may benefit from objective imaging‐based scar assessment and larger randomized controlled designs.

## Author Contributions

A.A., M.A.S., and A.M. contributed to the study conception and design. Data collection and analysis were performed by all authors. The first draft of the manuscript was written by R.E. and A.M., and all authors commented on previous versions of the manuscript. All authors read and approved the final manuscript.

## Funding

The authors have nothing to report.

## Ethics Statement

Reviewed and approved by Zagazig University‐ Institutional Review Board (ZU‐IRB # 9489‐13‐4‐2022).

## Conflicts of Interest

The authors declare no conflicts of interest.

## Data Availability

The data that support the findings of this study are available from the corresponding author upon reasonable request.
